# Swaddle bath vs. tub bath for physiological outcomes and skin microbiota in late preterm infants: a randomized controlled trial

**DOI:** 10.3389/fped.2025.1700944

**Published:** 2025-11-03

**Authors:** Xiaoqin Jia, Yihong Wu, Zhaomei Huang, Wanying Fan, Jun Chen, Xiudan Huang, Huihua Feng

**Affiliations:** Department of Neonatology, The Affiliated Foshan Women and Children Hospital, Guangdong Medical University, Foshan, China

**Keywords:** swaddle bath, tub bath, physiological outcomes, skin microbiota, late preterm infants, randomized controlled trial

## Abstract

**Objective:**

To explore the effects of two bathing methods, swaddle bath and tub bath, on physiological parameters and skin microbiota in late preterm infants.

**Design:**

Prospective, no-blinded, randomised controlled trial.

**Setting:**

Neonatal Intensive Care Unit in Foshan.

**Methods:**

56 late preterm infants were randomly divided into two groups: Intervention Group (swaddle bath, *n* = 28), control group (tub bath, *n* = 28). Physiological parameters, hemodynamics, stress responses and skin colony counts were evaluated pre(T_0_), immediate(T_1_), 30 min after bathing(T_2_).

**Results:**

The mean changes of rectal temperature, heart rate, respiration, and perfusion index (PI) in the intervention group were significantly higher than that of the control group immediately after bathing. The swaddle bath group showed less stress during the bathing process compared to the tub bath group (Crying: 1 vs. 8, *p* = 0.03, Clenched hands: 4 vs. 11, *p* = 0.04). No difference was found between the two groups, in terms of different peripheral oxygen saturation (SpO_2_), pulse variability index (PVI), or the number of skin colonies.

**Conclusion:**

Swaddle bath is a more recommended bathing for late preterm infants, as it ensures stable vital signs and blood perfusion while reducing stress manifestations during the bathing process.

**Clinical Trial Registration:**

https://www.chictr.org.cn/indexEN.html, identifier ChiCTR2400087426.

## Introduction

Bathing is a routine practice in the Neonatal Intensive Care Unit (NICU), serving as a cornerstone for the neonatal development, infection prevention, and the fortification of the epidermal barrier (1, 2). The World Health Organization has determined the normal range of neonatal body temperature as 36.5°C–37.5°C (3). Late preterm infants (34–36^+6^ weeks) are particularly vulnerable to thermal loss and infection due to their thin skin and compromised thermoregulation (4, 5). This population was prioritized to evaluate interventions that minimize stress and maintain physiological stability during routine care activities. Bathing exerts a protective effect on the skin barrier and inhibits bacterial colonization (6). During bathing, hypothermia or increased stress response may occur, and even serious complications such as hypoglycemia, respiratory arrest, metabolic acidosis, etc. (7, 8).

Previous studies have shown that immersion baths are beneficial for late preterm infants, especially swaddle bath (9). Swaddle Bath is a simulated intrauterine curled up and bent position for newborns, wrapped in a soft towel and immersed in warm water (38℃–40℃). Each limb, trunk, etc. of the body is gradually exposed separately for cleaning and rewrapping, and the newborn remains in a fixed midline position during the bathing period (10–12). Few studies to date have explicitly compared the effects of swaddle bath vs. traditional tub bath on physiological parameters and stress responses in late preterm infants.

This study was designed to conduct swaddle bath on late preterm infants in NICU. Exploring whether swaddle bath is superior to traditional tub bath in maintaining stable vital signs, reducing hemodynamic changes, and stress responses in late preterm infants, in order to clarify the impact of swaddle bath on late preterm infants.

• H1: Swaddle bath will better maintain physiological stability—including rectal temperature, heart rate, and respiratory rate—compared to tub bath.
• H2: Swaddle bath will decrease stress (e.g., PI changes, crying, Clenched hands).

## Methods

Research design: Prospective, no-blinded, randomised controlled trial.

**Figure 1:**
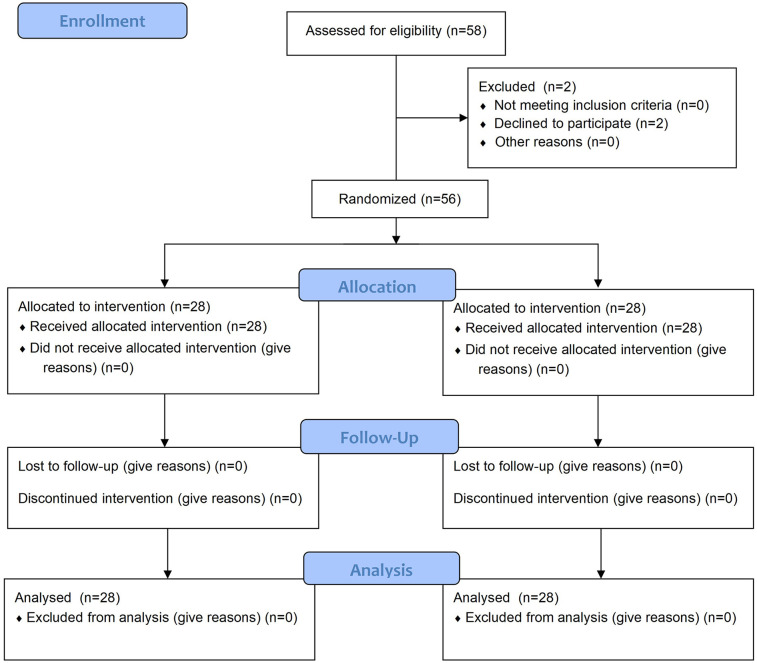
Study design.

Grouping: A computer-generated randomization program was utilized to assign newborns to either the intervention group (swaddle bath group) or the control group (tub bath group). A researcher, not involved in the study, will be responsible for allocation and data analysis. Participants were randomly allocated using computer-generated sequences created via *SPSS* 27.0. With *n* = 56 subjects numbered sequentially, odd-numbered participants formed the swaddling bath cohort and even-numbered counterparts constituted the traditional tub bath control group. Figure 1 illustrates the protocol according to Standard Protocol Items.

Research subjects: Preterm infants admitted to our NICU between July 2024 and November 2024 were recruited. All procedures were performed in NICU rooms maintained at 26°C–28°C with 50%–60% humidity. Inclusion: (1) 34–36^+6^ weeks gestational age at birth and birth >24 h; (2) Birth weight ≥1,500 g; (3) Apgar scores (≥8 at 5 min); (4) Stop feeding at least 1 h before bathing; (5) Informed consent obtained from parents. Exclusion: (1) Grade II–Ⅳ intraventricular hemorrhage; (2) Invasive mechanical ventilation or Nasal continuous positive airway pressure (NCPAP); (3) Hereditary diseases caused by mutations genes; (4) Neurometabolic disorders and congenital malformations of the central nervous system; (5) Using sedative and muscle-relaxant drugs. Elimination: (1) Severe clinical deterioration requiring urgent intervention (e.g., acute respiratory distress, shock); (2) Researcher's judgment, from a medical standpoint, that trial termination is necessary for the subject.

### Ethical approval

In accordance with the Declaration of Helsinki, we obtained ethical approval from the Affiliated Foshan Women and Children Hospital, Guangdong Medical University ethics committee for implementing this study (FSFY-MEC-2024-074).

Instrument: Electronic thermometer (MC-246, Omron Co., Ltd., China) measures rectal temperature. A pulse oximeter (RDS-3, Masimo Corp., Mexico) was employed for monitoring.

Outcome measures: The Preterm Infant Bathing Record Form includes general information (gestational age at birth, birth weight, gender, type of delivery, 5-min Apgar score, etc.) and outcome measurement indicators [including rectal temperature, heart rate, respiration, peripheral blood oxygen saturation, perfusion index (PI, perfusion index), variability index (PVI, pulse variability index), stress assessment, and number of skin colonies, etc.].

Data collection procedure: The measurement data were collected by the same research nurse. Rectal temperature served as the indicator of body temperature for preterm infants, recorded in degrees Celsius (℃). Respiratory rate was measured using a stopwatch over a 1-min period. Multi-parameter monitoring sensors were placed on the feet of preterm infants to measure heart rate, peripheral blood oxygen saturation, PI and PVI. Stress responses were identified through behaviors such as crying, clenched hands or resisting. Assessments occurred at three time points: before bathing (T_0_), immediately after bathing (T_1_), and 30 min after bathing (T_2_) (13). The number of skin colonies was assessed in both groups before and after bathing. Chest skin swabs were collected from each infant for cultivation to determine the total bacterial colony count, with fungi and pathogenic species excluded from the analysis.

Control group (14): (1) Operator Requirements: Experienced neonatal nurses (with at least one year of service) performed the procedure, ensuring clean and warm hands. (2) Environment and Materials: Room temperature was maintained at 26℃–28℃, relative humidity at 50%–60%, and lighting was kept soft (illuminance <100 lux). Equipment included a baby bathtub, thermometer, gauze towels, diapers, wet wipes, 0.9% normal saline, and disinfected cotton swabs. Water temperature was set at 40℃, with a depth of 10–12 cm, filling 1/2–2/3 of the bathtub. All infants had ceased breastfeeding at least 1 h prior to bathing. (3) Tub Bath Procedure: The operator washed hands thoroughly (with hand sanitizer and water for 40–60 s), then held the infant while washing the eyes and face in a conventional sequence. The left arm supported the infant's neck and back, with the buttocks nestled under the nurse's armpit, while the right hand washed and dried the infant's head. For the body wash, the nurse supported the infant with both hands, gradually lowering it into the water. The left hand stabilized the head and neck, allowing the infant to sit comfortably in the tub, with a small amount of water poured onto the chest for relaxation. The body was washed in the order of contralateral limbs, proximal limbs, chest and abdomen, back, and buttocks. Post-washing, the infant was thoroughly dried, received hip and umbilical cord care, and was then placed in an infant incubator.

**Figure 2:**
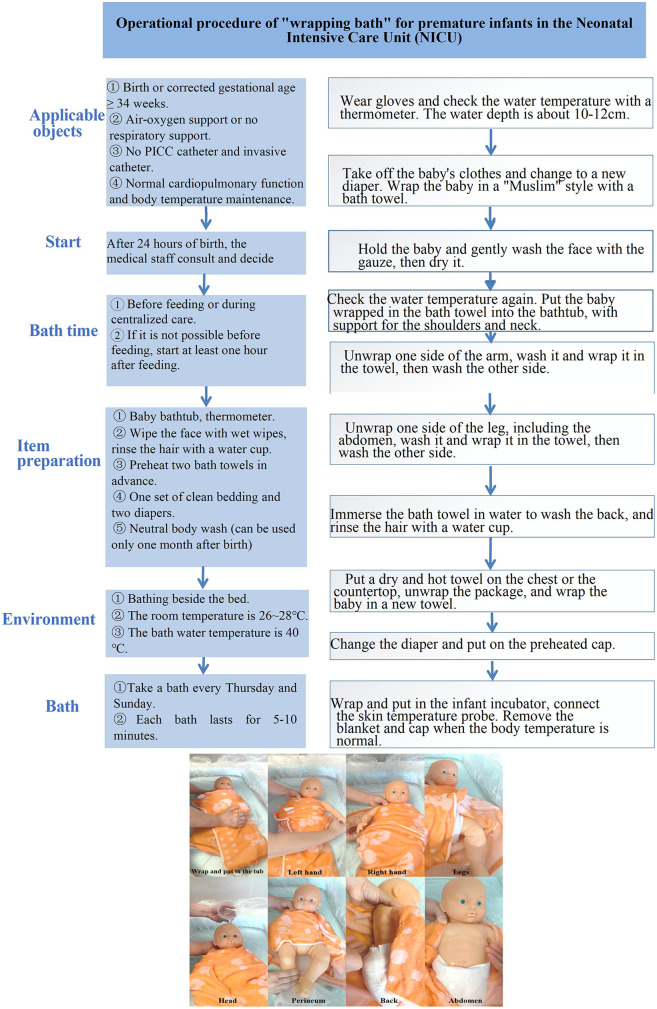
The operation process of swaddle bath.

Intervention group (15): (1) Operator Requirements, Environmental Preparation, and Equipment: Identical to the control group. (2) Swaddle Bath Procedure (Figure 2): Prior to water immersion, the infant was wrapped in a bath towel using the swaddling technique to maintain a flexed midline position, preserving the natural curvature of the trunk, arms, and legs. The infant was then slowly immersed in warm bathtub water until the water level reached the shoulders, ensuring foot contact with the tub bottom. During bathing, the swaddling layers were progressively opened according to the washing sequence, with re-covering after each side was cleaned. Post-bathing, the wet bath towel was removed, and the infant was transferred to a dry towel for thorough drying and hip/umbilical cord care. The infant was then wrapped in a towel and placed in an incubator, with the towel removed after 30 min.

### Statistical analysis

Data will be evaluated descriptively (mean, standard deviation, and median for metric variables, frequency and percentiles for categorical variables) for all variables as available case analysis. The descriptive analysis will be presented as measures of central tendency and dispersion. The *t*-test for independent samples or the Mann–Whitney U test will be used for intergroup comparisons. Repeated measures ANOVA and generalized estimating equations (GEE) accounted for within-subject changes over time.

## Results

A total of 56 late preterm infants were screened for eligibility. All met the inclusion criteria and were randomized into either the swaddle bath group (*n* = 28), or the tub bath group (*n* = 28). The groups were well balanced for demographic and baseline characteristics (Table 1).

**Table 1:** Demographic and baseline characteristics of study participants (*N* = 56).

Variable	Swaddle bath group (*n* = 28)	Tub bath group (*n* = 28)	*t*/*χ*^2^	*p*
Gestational age^a^ (weeks, mean ± SD)	35.05 ± 0.91	34.91 ± 0.95	0.16	0.57
Maternal Age^a^ (years, mean ± SD))	33.39 ± 3.74	33.75 ± 3.22	0.33	0.70
Birth weight^a^ (g, mean ± SD)	2,515.00 ± 390.72	2,503.00 ± 323.33	1.06	0.89
Gender^b^ (*n*, %)			1.14	0.29
Male	12 (42.90)	16 (57.10)		
Female	16 (57.10)	12 (42.90)		
Type of delivery^b^ (*n*, %)			0.29	0.60
Vaginal	12 (42.90)	16 (57.10)		
Cesarean birth	14 (50.00）	14 (50.00）		

Mean ± SD = mean ± standard deviation, *t* = *t*-test, *χ*^2^ = chi-square test.

^a^ Two-sample *t*-test.

^b^ Chi-square test.

Rectal temperature after bathing was significantly higher for the swaddle bath group vs. tub bath group (*p* < 0.001, Table 2). The results of repeated measurements showed that there were statistically significant differences in the time effect, main effect, and interaction effect, indicating that the changing trend of the rectal temperature of infants over time was different under the two bathing methods. After bathing, the rectal temperature of infants in the swaddle bath group was more stable than that in the tub bath group. The differences in heart rate and respiratory rate between the two groups after bathing were significant (HR: *p* = 0.02, RR: *p* = 0.03), but repeated measurement results showed that the main effects of these were not statistically significant. There was no significant difference in the blood oxygen saturation between two groups immediately after bathing (swaddle vs. tub bath, *p* = 0.85). The repeated measurements showed that only the time effect was statistically significant.

**Table 2:** Comparison of physiological parameters between the swaddle bath and tub bath groups (mean ± SD).

Variable	Swaddle bath group (*n* = 28)	Tub bath group (*n* = 28)	*t*	*p*	Time effect	Group effect	Group ×Time effect
Temperature (◦C)							
before bathing	37.10 ± 0.15	37.06 ± 0.14	0.81^1^	0.42			
Immediately after bathing	36.99 ± 0.14	36.79 ± 0.15	4.85^1^	<0.001**			
30 min after bathing	37.06 ± 0.13	37.04 ± 0.16	0.37^1^	0.72			
F					101.62	5.04	23.82
*p*					<0.001**	0.03*	<0.001**
Heart rate (per minute)							
before bathing	144.57 ± 4.04	144.54 ± 5.07	0.03^1^	0.98			
Immediately after bathing	141.11 ± 3.99	138.71 ± 3.40	2.42^1^	0.02*			
30 min after bathing	144.00 ± 3.42	144.29 ± 2.79	0.34^1^	0.73			
F					50.95	0.72	4.12
*p*					<0.001**	0.40	0.03*
Respiratory rate(per minute)							
before bathing	46.86 ± 1.43	46.46 ± 1.37	1.05^1^	0.30			
Immediately after bathing	45.07 ± 1.30	44.04 ± 1.23	3.06^1^	0.003*			
30 min after bathing	46.43 ± 1.23	46.36 ± 1.13	0.23^1^	0.82			
F					58.57	4.28	2.68
*p*					<0.001**	0.04*	0.08
Oxygen saturation (%)							
before bathing	98.07 ± 1.33	97.93 ± 1.21	0.42^1^	0.68			
Immediately after bathing	97.25 ± 1.38	97.18 ± 1.36	0.20^1^	0.85			
30 min after bathing	97.93 ± 1.36	97.71 ± 1.21	0.62^1^	0.54			
F					8.68	0.29	0.07
*p*					0.001**	0.59	0.91

Mean ± SD = mean ± standard deviation, F = F-statistic from repeated measures ANOVA, *t* = *t*-test.

Time effects refer to changes over time within groups; group effects indicate between-group differences; group × time interactions reflect differential responses across groups. Mixed-model analysis accounted for repeated measurements.

^1^ Two-sample *t*-test.

* *p* < 0.05.

** *p* < 0.001.

No significant group differences in PI were detected before bathing (*p* = 0.09) and 30 min after bathing (*p* = 0.06) (Table 3). The PI of infants who underwent tub bath was slightly lower than that of those who underwent swaddle bath. The GEE analysis showed that there were significant differences in time effect and group effect, with no significant interaction effect (*p* = 0.51). There were no significant group differences in PVI values across different stages. GEE showed no significant group effect, but significant time and interaction effects, indicating similar trends in PVI changes across different stages in both groups.

**Table 3:** Comparison of perfusion index and pleth variability index between the swaddle bath and tub bath groups [M (IQR)].

Variable	Group	before bathing	Immediately after bathing	30 min after bathing	*χ* ^2^	*P*
PI (%)	Swaddle bath group (*n* = 28)	1.90 (0.67)	1.70 (0.53)	2.00 (0.50)	27.83	<0.001**
	Tub bath group (*n* = 28)	1.80 (0.45)	1.40 (0.18)	1.85 (0.30)	27.83	<0.001**
	Z	1.71	2.50	1.90		
	*p*	0.09	0.01*	0.06		
	Wald χ*2* group = 7.09; Wald χ^2^ time = 164.27; Wald χ*2* group*time = 1.36 *p* group = 0.01; *p* time<0.001; *p* group*time = 0.51					
PVI (%)	Swaddle bath group (*n* = 28)	16.00 (3.00)	19.00 (4.00)	16.5 (2.00)	30.08	<0.001**
	Tub bath group (*n* = 28)	17.00 (3.00)	19.00 (2.00)	17.00 (3.00)	22.94	<0.001**
	Z	1.16	0.52	1.12		
	*p*	0.25	0.61	0.26		
	Wald *χ*^2^ group = 0.44; Wald *χ*^2^ time = 136.88; Wald *χ*^2^ group*time = 8.89 *p* group = 0.51; *p* time <0.001; *p* group*time = 0.01					

PI, perfusion index; PVI, pulse variability index; M, median; IQR, interquartile range; *χ*^2^, chi-square test.

Data are presented as M (IQR). Significant changes in PI over time were detected via *χ*^2^ tests. PVI trends showed no significant group differences.

* *p* < 0.05.

**p < 0.001.

The Swaddle bath group had fewer newborns who cried (*p* = 0.03) or clenched their hands (*p* = 0.04) compared to the tub bath group, while there was no difference in resistance (*p* = 0.53) (Table 4).

**Table 4:** Comparison of the percentage of crying, scratching, resisting, and colony numbers between the two groups.

Variable	Swaddle bath group (*n* = 28)	Tub bath group (*n* = 28)	χ^2^/*t*	*p*
Crying	1 (3.60)	8 (28.60)	4.77	0.03*
Clenched hands	4 (14.29)	11 (39.29)	4.46	0.04*
Resisting	4 (14.29)	6 (21.43)	0.08	0.53
Skin microbiota	777.56 ± 590.80	506.00 ± 427.90	1.07	0.30

*t* = *t*-test, *χ*^2^ = chi-square test.

* *p* < 0.05.

The number of skin colonies in infants decreased after bathing in both groups, with no significant difference in the change in colony numbers between the two groups (*p* = 0.30), Table 4.

## Discussion

In our study, we investigated the effects of two bathing methods, swaddle bath and tub bath, on the physiological parameters and skin microbiota in late preterm infants. We found that these two methods had a significant impact on several key physiological parameters such as rectal temperature, heart rate, respiratory rate, perfusion index (PI), and stress-related behaviors (e.g., crying and clenching hands).

Bathing is known to cause heat loss from the skin, particularly in late preterm infants who are more susceptible to temperature regulation issues due to their delicate skin (16). Both bathing methods led to a decrease in body temperature, with the tub bath group experiencing a more pronounced decrease than the swaddle bath group. The body temperature stability of the swaddle bath group was better than that of the tub bath group. This finding aligns with previous research by Huang et al. (17), which suggests that the reduced heat evaporation loss during the swaddle bath process contributes to better temperature stability.

Both bathing methods caused immediate changes in heart rate, respiratory rate, and blood oxygen saturation after the bath. A large number of studies have shown that bathing stimulation of infants can lead to instability of these parameters (17, 18). In our study, the decrease in heart rate and respiratory rate immediately after bathing in the swaddle bath group was smaller than that in the tub bath group. However, there was no statistically significant difference in heart rate and respiratory rate 30 min after bathing in both groups. This may be related to the decrease in heart rate and respiratory rate caused by heat dissipation during bathing. Additionally, there was no statistically significant difference in blood oxygen saturation between the two groups, indicating that both bathing methods had certain reliability in maintaining the stability of blood oxygen saturation in preterm infants. However, it is worth noting that changes in blood oxygen saturation may be affected by multiple factors, such as the health status of preterm infants and the bathing environment, etc. (19).

The PI can be used to assess peripheral perfusion in preterm infants (20, 21). Some studies have found that PI measurement is affected by cardiac output and the state of the sympathetic nervous system. The latter can be influenced by factors such as intravenous injection therapy, bathing, and awakening state (22). Our study showed that both bathing methods led to a transient decrease in PI immediately after bathing, followed by a gradual recovery. The changes in PI before bathing, immediately after bathing and 30 min after bathing were more stable in the swaddle bath group than in the tub bath group. We believe that the swaddle bath may have a positive impact on the state of preterm infants by wrapping the infants to reduce the stress response. PVI is affected by the behavioral state of infants (23, 24). There was no significant difference in the PVI values between the two bathing methods in our study. This might be related to factors such as the small sample size and the short observation time in this study.

Caka et al. (25) found that swaddle bathing can keep preterm infants in a calm state by reducing crying and restlessness. In our study, 8 infants cried in the tub bath group, compared to only one in the swaddle bath group. Premature infants in the swaddle bath group also showed fewer signs of tension, such as reduced hand clenching. This may be related to the sense of enclosure and security provided by swaddle bathing (26). Both groups of late preterm infants showed less resistance, possibly because both bathing methods were performed in a basin, eliminating the stimulation from flowing water (27).

In the short term, swaddle bathing appears to reduce thermal instability and stress-related behaviors, which are critical for late preterm infants who are particularly vulnerable to hypothermia and autonomic dysregulation. These immediate benefits may contribute to improved neurodevelopmental outcomes over time, as repeated exposure to stress and thermal instability in the neonatal period has been associated with adverse long-term effects, including altered stress response systems and neurodevelopmental delays. Although our study did not assess long-term outcomes, the observed stabilization of vital signs and reduced stress responses suggest that swaddle bathing may have protective effects that warrant investigation in longitudinal studies.

Bathing is crucial for maintaining the optimal skin condition of preterm infants (28), as it can reduce bacterial colonization of the skin (6). We found that both bathing methods effectively reduced skin bacterial colonization (as indicated by similar colony counts), suggesting comparable cleaning efficacy. Nevertheless, swaddle bath is preferable due to its additional benefits in stabilizing body temperature and mitigating stress responses.

## Limitations

This study has limitations, including a small sample size, which necessitate further validation of certain findings. Additionally, the lack of blinding for caregivers or data collectors may introduce observer bias, potentially affecting the objectivity of outcome assessments (e.g., physiological measurements, stress behaviors). Another limitation is that all physiological and behavioral data were collected by a single research nurse. While this ensured consistency in data collection procedures, it may also introduce observer bias, particularly in the assessment of stress-related behaviors. Future studies should consider involving multiple trained data collectors and implementing inter-rater reliability assessments to minimize this potential bias. Future trials should therefore recruit multiple trained observers, document inter-rater reliability, implement double-blind designs, enlarge the sample, prolong observation periods, and systematically explore the long-term health impacts of swaddle bath on late preterm infants.

## Conclusion

Our findings suggest that swaddle bath may be preferable to tub bath in late preterm infants, but larger multicenter trials are needed to confirm these results. These findings offer novel insights for NICU nursing practice and may improve both short- and long-term health outcomes in preterm infants.

## Data Availability

The raw data supporting the conclusions of this article will be made available by the authors, without undue reservation.

## References

[1] Burdall O Willgress L Goad N. Neonatal skin care: developments in care to maintain neonatal barrier function and prevention of diaper dermatitis. Pediatr Dermatol. (2019) 36(1):31–5. 10.1111/pde.13714 30506880

[2] Soltani N Seyedrasooli A Jabraeili M Mousavi S. The effect of maternal multisensory stimulations on bath stress in premature infants: a randomized controlled clinical trial. Infant Behav Dev. (2022) 67:101720. 10.1016/j.infbeh.2022.101720 35561627

[3] World Health Organization. Maternal and Newborn Health, Thermal Protection of the Newborn: A Practical Guide. Geneva, Switzerland: World Health Organization (1997). p. 64.

[4] Evidence-Based, M.G., S. Neonatologist and M.D.A. Chinese, [guidelines for neonatal skin management in the neonatal intensive care unit (2021)]. Zhongguo Dang Dai Er Ke Za Zhi. (2021) 23(7):659–70. 10.7499/j.issn.1008-8830.2106004 34266521 PMC8292657

[5] Altay G Kucukoglu S. Effects of the facilitated tucking position in early period on physiological parameters, comfort and breastfeeding performance in late preterm infants: a randomized controlled trial. Midwifery. (2022) 115:103492. 10.1016/j.midw.2022.103492 36201966

[6] Kusari A Han AM Virgen CA Matiz C Rasmussen M Friedlander SF Evidence-based skin care in preterm infants. Pediatr Dermatol. (2019) 36(1):16–23. 10.1111/pde.13725 30548578

[7] Andrews C Whatley C Smith M Brayton EC Simone S Holmes AV. Quality-improvement effort to reduce hypothermia among high-risk infants on a mother-infant unit. Pediatrics. (2018) 141(3):e20171214. 10.1542/peds.2017-1214 29444816

[8] World Health Organization. Pregnancy, Childbirth, Postpartum and Newborn Care: A Guide for Essential Practice. Geneva: World Health Organization (2016). 26561684

[9] Ar I Gozen D. Effects of underrunning water bathing and immersion tub bathing on vital signs of newborn infants: a comparative analysis. Adv Neonatal Care. (2018) 18(6):E3–12. 10.1097/ANC.0000000000000484 30507829

[10] Göçmen G Ek şio ğlu A Oran NT Çeber E. Effects of sponge bath and swaddle bath applied in the neonatal intensive care unit on neonatal comfort: a randomized controlled trial. J Pediatr Nurs. (2025) 84:381–9. 10.1016/j.pedn.2025.06.048 40639057

[11] Fernandez D Antolin-Rodriguez R. Bathing a premature infant in the intensive care unit: a systematic review. J Pediatr Nurs. (2018) 42:e52–7. 10.1016/j.pedn.2018.05.002 29779763

[12] Lund C. Bathing and beyond: current bathing controversies for newborn infants. Adv Neonatal Care. (2016) 16(Suppl 5S):S13–20. 10.1097/ANC.0000000000000336 27676109

[13] de Freitas P Bueno M Holditch-Davis D Santos HP Kimura AF. Biobehavioral responses of preterm infants to conventional and swaddled tub baths: a randomized crossover trial. J Perinat Neonatal Nurs. (2018) 32(4):358–65. 10.1097/JPN.0000000000000336 29782435

[14] Zhang Y Wang Y. Pediatric Nursing (5th Edition/Higher Vocational Nursing/Added Value) Chinese Ed. Beijing: People’s Health Publishing House (2024). p. 197.

[15] BabyDam. What is a swaddle bath? BabyDam website. Published: 24th June 2019. Available online at: https://babydam.com/what-is-a-swaddle-bath/ (Accessed August 17, 2023).

[16] Tasdemir HI Efe E. The effect of delaying first bathing on skin barrier function in late preterm infants: a study protocol for multi-centre, single-blind RCT. J Adv Nurs. (2021) 77(2):1051–61. 10.1111/jan.14657 33210328

[17] Huang Y Zhou L Abdillah H Hu B Jiang Y. Effects of swaddled and traditional tub bathing on stress and physiological parameters of preterm infants: a randomized clinical trial in China. J Pediatr Nurs. (2022) 64:e154–8. 10.1016/j.pedn.2021.11.028 34953663

[18] Cinar N Yalnizoglu CS Uslu YH. Effect of newborn bathing training with the swaddled and tub bathing methods given to primiparous pregnant women on the mother’s experience, satisfaction and newborn’s stress during the first bathing of the newborn at home: a mixed method study. Jpn J Nurs Sci. (2020) 17(4):e12363. 10.1111/jjns.12363 32844590

[19] Tasdemir HI Efe E. The effect of tub bathing and sponge bathing on neonatal comfort and physiological parameters in late preterm infants: a randomized controlled trial. Int J Nurs Stud. (2019) 99:103377. 10.1016/j.ijnurstu.2019.06.008 31442786

[20] Mandala VK Mendu SB Bollaboina SKY Kotha R Sr. Role of perfusion index and pulse variability index in the assessment of neonatal hemodynamics: a systematic review. Cureus. (2023) 15(10):e48058. 10.7759/cureus.48058 38046508 PMC10688761

[21] Zhao Y Yang G Niu S Zhang M Gao F Liu K. Evaluation of tissue perfusion status in moderate to late preterm. Physiol Res. (2022) 71(5):607–14. 10.33549/physiolres.934888 36047728 PMC9841808

[22] Cresi F Pelle E Calabrese R Costa L Farinasso D Silvestro L. Perfusion index variations in clinically and hemodynamically stable preterm newborns in the first week of life. Ital J Pediatr. (2010) 36:6. 10.1186/1824-7288-36-6 20205908 PMC2828459

[23] Fischer MO Lemoine S Tavernier B Bouchakour CE Colas V Houard M Individualized fluid management using the pleth variability index: a randomized clinical trial. Anesthesiology. (2020) 133(1):31–40. 10.1097/ALN.0000000000003260 32205547

[24] Cistone N Pickler RH Fortney CA Nist MD. Effect of routine nurse caregiving on the stress responses and behavior state in preterm infants: a systematic review. Adv Neonatal Care. (2024) 24(5):442–52. 10.1097/ANC.0000000000001177 38968382 PMC11361837

[25] Caka SY Gozen D. Effects of swaddled and traditional tub bathing methods on crying and physiological responses of newborns. J Spec Pediatr Nurs. (2018) 23(1). 10.1111/jspn.12202 29160925

[26] Pereira S. Newborns’ behavioral adaptations during hot tub bath: a randomized clinical trial. J Pediatr Neonatal Care. (2017) 6. 10.15406/jpnc.2017.06.00245

[27] Sun X Xu J Zhou R Liu B Gu Z. Effectiveness of different bathing methods on physiological indexes and behavioral status of preterm infants: a systematic review and meta-analysis. BMC Pediatr. (2023) 23(1):507. 10.1186/s12887-023-04280-y 37828460 PMC10571243

[28] Lee JC Lee Y Park HR. Effects of bathing interval on skin condition and axillary bacterial colonization in preterm infants. Appl Nurs Res. (2018) 40:34–8. 10.1016/j.apnr.2017.12.012 29579496

